# Perseverative Cognition and Health Behaviors: A Systematic Review and Meta-Analysis

**DOI:** 10.3389/fnhum.2016.00534

**Published:** 2016-11-08

**Authors:** Faye Clancy, Andrew Prestwich, Lizzie Caperon, Daryl B. O'Connor

**Affiliations:** ^1^School of Psychology, University of LeedsLeeds, UK; ^2^Leeds Institute of Health Sciences, University of LeedsLeeds, UK

**Keywords:** stress, worry, rumination, alcohol, smoking, exercise, diet, health

## Abstract

Recent developments in stress theory have emphasized the significance of perseverative cognition (worry and rumination) in furthering our understanding of stress-disease relationships. Substantial evidence has shown that perseverative cognition (PC) is associated with somatic outcomes and numerous physiological concomitants have been identified (i.e., cardiovascular, autonomic, and endocrine nervous system activity parameters). However, there has been no synthesis of the evidence regarding the association between PC and health behaviors. This is important given such behaviors may also directly and/or indirectly influence health and disease outcomes (triggered by PC). Therefore, the aim of the current review was to synthesize available studies that have explored the relationship between worry and rumination and health behaviors (health risk: behaviors which, if performed, would be detrimental to health; health promoting: behaviors which, if performed, would be beneficial for health). A systematic review and meta-analyses of the literature were conducted. Studies were included in the review if they reported the association between PC and health behavior. Studies identified in MEDLINE or PsycINFO (*k* = 7504) were screened, of which 19 studies met the eligibility criteria. Random-effects meta-analyses suggested increased PC was generally associated with increased health risk behaviors but not health promoting behaviors. Further analyses indicated that increases in rumination (*r* = 0.122), but not reflection (*r* = −0.080), or worry (*r* = 0.048) were associated with health risk behaviors. In conclusion, these results showed that increases in PC are associated with increases in health risk behaviors (substance use, alcohol consumption, unhealthy eating, and smoking) that are driven primarily through rumination. These findings provide partial support for our hypothesis that in Brosschot et al.'s (2006) original perseverative cognition hypothesis, there may be scope for additional routes to pathogenic disease via poorer health behaviors.

## Introduction

In 2006, Brosschot, Gerin, and Thayer introduced the perseverative cognition hypothesis (PCH), which suggested that worry and/or repetitive thinking may lead to disease by prolonging stress-related physiological activation by amplifying short-term responses, delaying recovery, or reactivating responses after a stressor has been experienced. In the last decade, a number of important reviews and papers have been published clearly demonstrating that **perseverative cognition** is associated with somatic outcomes (e.g., Brosschot et al., 2005; Verkuil et al., 2010; O'Connor et al., 2013; Ottaviani et al., 2015).

More specifically, the PCH proposes that worry, rumination and related thought processes are not only psychological phenomena but can also impact on physical health. It is argued that perseverative cognition (PC)—the cognitive representation of past stressful events or feared future events—mediates the relationship between stress and physical disease as, when stressors are perseverated upon in thought, the damaging physiological activation associated with stress is also protracted, thus increasing susceptibility to stress-related ill-health. The hypothesis states that, in such instances where the physical stressor is absent, the cognitive representation alone can induce a physiological stress response, which, when prolonged, increases the likelihood of stress-related diseases. In this sense, the direct relationship between stress and disease is intensified when a stressor is subject to thought.

Since the PCH was proposed, a substantial amount of evidence has been identified which supports the main tenets of the theory. In one of the first reviews published, Verkuil et al. (2010) presented convincing research evidence of a link between the prolonged physiological activation associated with PC and somatic health outcomes. More recently, Ottaviani et al. (2015) conducted a comprehensive meta-analysis to synthesize the physiological concomitants of PC in healthy participants. These authors concluded that there was clear evidence that PC affects cardiovascular, autonomic, and endocrine nervous system pathways consistent with a pathogenic route to long-term disease outcomes. Specifically, they found higher levels of heart rate, blood pressure, and cortisol activity and lower heart rate variability during PC or related to trait PC.

However, despite the accumulating evidence for a direct pathway from PC to disease outcomes, we were interested in exploring the existence of an additional indirect pathway via health behaviors. In the broader stress literature, it is well-established that stress can affect health indirectly, through the modification of health behaviors (Rod et al., 2009; O'Connor and Conner, 2011). Stress induced modifications of habitual health behaviors such as food choice and eating behavior have been shown to be particularly important in understanding physical disease risk (Steptoe et al., 1998; O'Connor et al., 2008). Recent findings have confirmed that stress is frequently associated with increased unhealthy food intake in laboratory-based and naturalistic studies (e.g., Adam and Epel, 2007; O'Connor et al., 2008; Dallman, 2010; Van Strien et al., 2012).

For example, in a 28-day diary study, O'Connor et al. (2008) showed that daily stressors were associated with increased consumption of high fat and high sugar between-meal snack foods and with a reduction in main meals and vegetable consumption. Moreover, evidence is beginning to emerge showing associations between rumination and the consumption of unhealthy foods such as cakes, crisps, and confectionary (e.g., Cropley et al., 2012). Therefore, it remains possible that PC might also amplify, prolong, and reactivate the same physiological and psychological processes that account for the negative effects of stress on eating behavior.

Other studies have provided evidence of a relationship between stress and increased alcohol consumption, which has been identified as a significant risk factor for chronic disease (Rehm et al., 2009). For example, in a daily diary study, Grzywacz and Almeida (2008) reported that participants were more likely to binge drink on days when they experienced more severe stressors. Similarly, in an experimental study, a blunted cortisol response to a laboratory stressor was associated with greater post-stressor alcohol consumption (Pratt and Davidson, 2009). Corbin et al. (2013) suggest that alcohol may be used to deal with negative emotion when alternative coping strategies are not available. In the sample of college students they surveyed, stress levels were positively associated with drinking to cope, and drinking problems. Moreover, those who reported drinking to cope drank more heavily. Again, similar to eating behavior, data are emerging showing that measures of (negative work) rumination are associated with more alcohol consumption on workdays (Frone, 2015).

**Figure 1:**
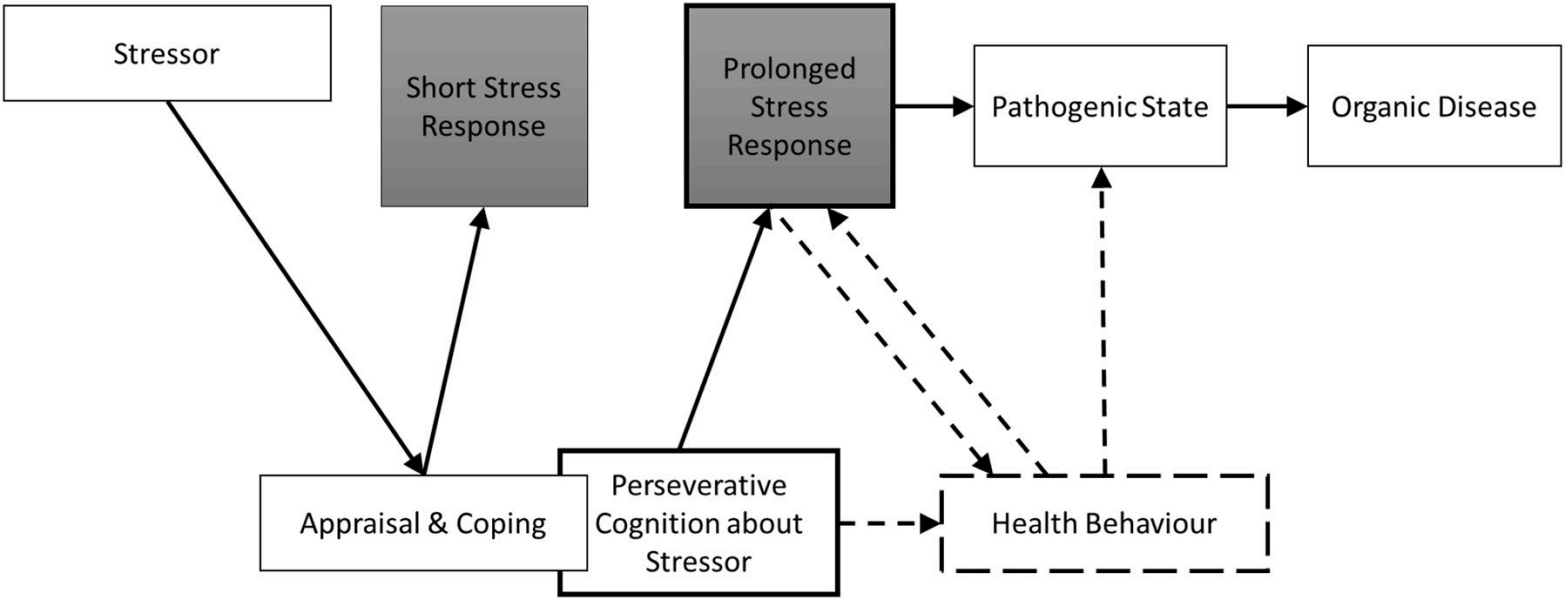
**Brosschot et al.'s (2006) model of PC and health extended to include additional pathways to illness via health behaviors (represented by dashed lines)**. PC may mediate the negative effects of stressors on health behaviors that will influence the pathogenic state (pathway #1). PC may also influence health behaviors through its effects on the prolonged stress response (pathway #2) and health behaviors may also have a bi-directional influence on the prolonged stress response (pathway #3).

Nevertheless, taking the above findings together, it is surprising how little research has explicitly explored the relationship between measures of PC and health behaviors. In addition, it is important to distinguish between health promoting and health risk behaviors. Health-promoting behaviors are health-enhancing behaviors which individuals are encouraged to perform more to protect their health; whereas health risk behaviors are health-damaging behaviors which individuals are encouraged to perform less. Given that PC exacerbates the relationship between the experience of stress and the physiological response, it is also possible that, as the experience of the stressor is prolonged by worry, or ruminative processes, so too may be its detrimental impact on different types of health behaviors. For example, PC might be more strongly associated with health risk behaviors such as alcohol consumption, smoking, and high fat food intake compared to health promoting behaviors such a physical exercise, given the former may be strategies to help alleviate rumination, and worry. Furthermore, over time, PC-induced increases in health risk behaviors and decreases in health promoting behaviors are likely to influence pathogenic pathways to long-term disease outcomes. Figure 1 represents the original model proposed by Brosschot et al. (2006) with an additional route to the pathogenic disease state via poorer health behaviors (e.g., higher levels of alcohol, tobacco and unhealthy food consumption, and lower physical activity levels and lower consumption of healthy foods). In this conceptualization, we theorize that rumination about past stressful events or worry about feared future events will mediate the effects of stressors on health behaviors (particularly those previously shown to be influenced by stress), which will have negative consequences for health outcomes and disease processes. Therefore, the primary aim of the current review and meta-analysis was to quantify the existing evidence relating any measure of PC to health behaviors.

A secondary aim of the current review and meta-analysis was to establish whether different types of PC had a differential impact on health behaviors. As outlined above, PC is an umbrella term which encompasses repetitive, negative thought processes related to the experience of a stressor. This term was developed as it was thought that disparate concepts such as rumination and worry were either too narrowly or too broadly defined to allow for a model which linked negative, repetitive thought, and somatic health (Verkuil et al., 2010). Indeed, there has been recent debate about whether rumination and worry ought to be considered separately or collapsed into a single phenomenal category (cf., Ottaviani et al., 2015). Nevertheless, the most widely researched of these thought processes are depressive rumination and worry (Verkuil et al., 2010). Nolen-Hoeksema et al. (2008) described rumination as “thinking perseveratively about one's feelings and problems” (p. 400) regardless of thought content (positive or negative). However, although ruminative thoughts can be positive, within the PCH, PC only encompasses negative thoughts (Verkuil et al., 2010).

Moreover, there is good agreement that rumination is best conceptualized as having two components: brooding and reflection (Treynor et al., 2003). Brooding is described as a passive and judgemental form of rumination, whereas reflection is more contemplative with a focus on problem-solving. Treynor et al. (2003) provided evidence that brooding is the more maladaptive component of rumination as brooding predicted symptoms of depression one year later, whereas, although reflection predicted current depression, it predicted lower levels of depression over time. Reflection is thus considered to be a somewhat adaptive component of rumination.

Whereas rumination has been shown to be associated with depression, worry is a central aspect of anxiety disorders, and particularly generalized anxiety disorder (Borkovec and Inz, 1990). Borkovec et al. (1983) were the first research group to aim to define and categorize the process of worrying and to distinguish it from related processes such as anxiety, fear, and mental problem-solving. Borkovec et al. (1983) defined worry as “a chain of thoughts and images, negatively affect-laden, and relatively uncontrollable. The worry process represents an attempt to engage in mental problem-solving on an issue whose outcome is uncertain but contains the possibility of one or more negative outcomes. Consequently, worry relates closely to fear processes” (p. 10). Therefore, within the PCH, worry is viewed as worry about feared events (or stressors) in the future.

To summarize, the primary aim of the current review was to systematically review empirical studies which have investigated the relationship between any type of PC and any health behavior outcome. As the PCH aimed to model how stress-related thinking may impact on health outcomes in otherwise healthy populations, the aim here was also to review studies involving physically and mentally healthy participants. It was hypothesized that higher levels of PC would be associated with more health risk behaviors (defined as those behaviors which, if performed, would hinder health) and less health promoting behaviors (defined as those behaviors which, if performed, would benefit health). The secondary aim was to explore whether different types of PC (rumination and worry) had differential effects on health behaviors.

## Methods

### Eligibility criteria

**Figure 2:**
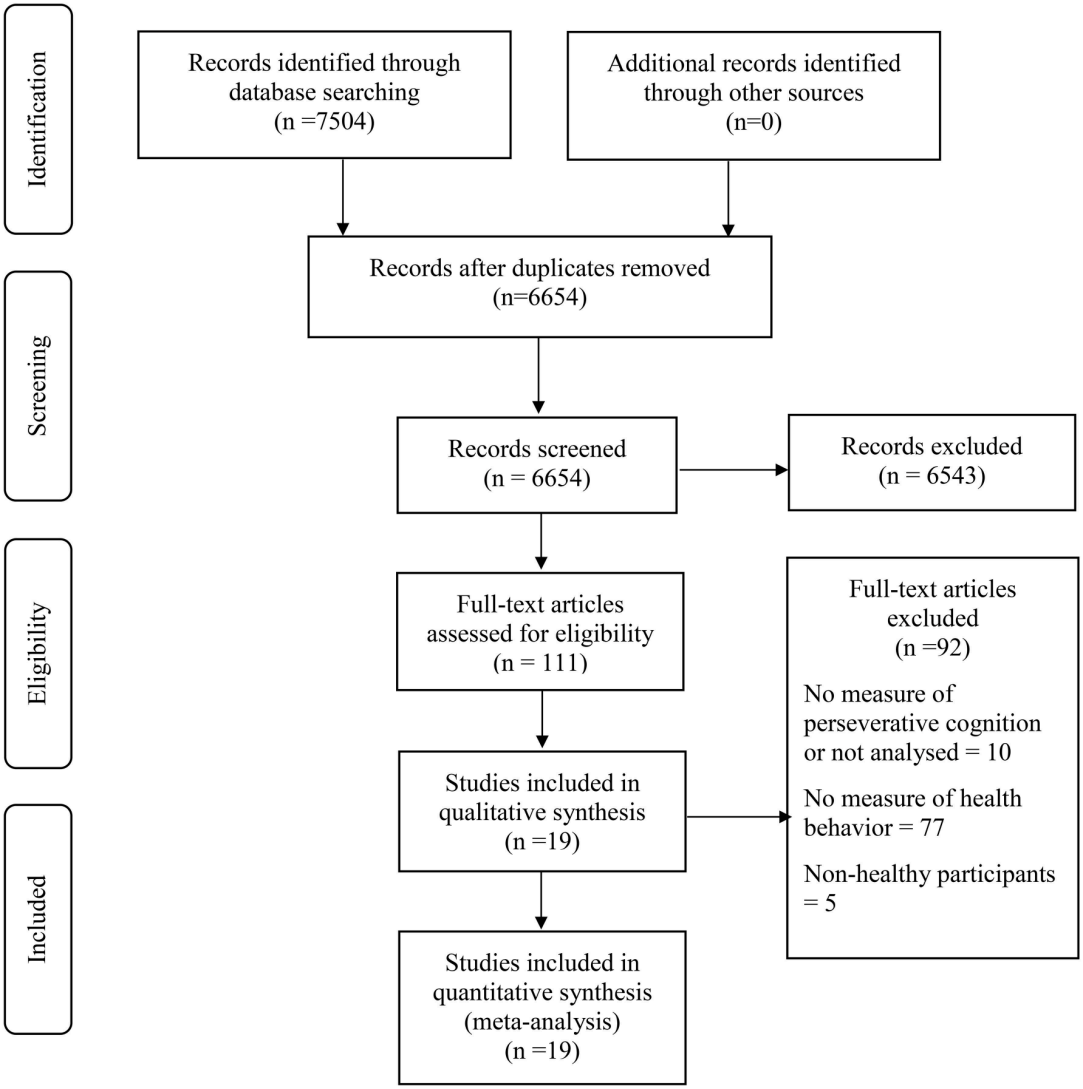
**PRISMA flow diagram depicting the screening process**. Figure adapted from Moher et al. (2009).

To be eligible, studies had to (1) include a measure of PC, (2) a measure of health behavior and, (3) report the relationship between the measures of PC and the health behavior within a statistical analysis that could be used to estimate an effect size (even if the relationship between PC and health behaviors was not the primary outcome of the study). Studies were excluded if they were (1) not peer-reviewed, (2) not an empirical investigation, (3) were reviews, editorials or “think pieces,” dissertations, book chapters, protocols, or unpublished, (4) if all study participants had been diagnosed with physical or mental health problems (but included if a sample of healthy participants was analyzed separately). Finally, studies that related to sleep (*n* = 75) were excluded from the current review paper (see Figure 2) because we considered sleep to be different from the other health behaviors under consideration. It is a complex behavior that is measured in many different ways and has multiple features (e.g., hours slept, sleep latency, sleep quality, insomnia, etc.) that sets it apart from behaviors such as smoking, physical activity, or eating. In addition, we felt that combining the relatively large number of sleep studies with the other health behaviors could potentially bias the results of the review. Therefore, given these points, the sleep studies will be synthesized in a separate review paper.

In terms of eligibility criterion (1), some researchers have argued that concepts such as angry rumination and co-rumination are separate forms of rumination. Angry rumination is a type of rumination in which the focus of the rumination is on an anger-inducing event and has been found to predict aggressive behavior (Denson, 2013) and was included in our conceptualization here. However, co-rumination is described as a group form of rumination in which interpersonal discussion focuses upon emotions and problems (Rose, 2002) but was not included here as it is not a purely cognitive form of PC (a similar approach was adopted by Ottaviani et al., 2015). Also, despite research which suggests that reflection may serve as an adaptive component of rumination, studies measuring reflection were retained in order to assess whether this type of rumination is still adaptive in terms of health behaviors (but analyzed separately from PC).

### Search strategy

PsycINFO (1806 to Present) and Medline (1946 to Present) were searched using OVID. The search was last run on the 11th of February 2016 using search terms relating to PC and health behavior. The search was limited by (1) English language, (2) human studies, and (3) studies published from 1990 [i.e., the year the Penn State Worry Questionnaire (Meyer et al., 1990) was published and shortly before the publication of key papers using the Ruminative Responses Scale (e.g., Nolen-Hoeksema, 1991)]. As with other systematic reviews, we wanted to strive for an appropriate trade-off between specificity (proportion of non-relevant articles that are not retrieved) and sensitivity (proportion of relevant articles that are retrieved). By restricting the search strategy to articles published from 1990 onwards (i.e., at the time several of our key measures were published), we anticipated a much greater increase in specificity with a relatively small reduction in sensitivity. Indeed, of the articles included in our review, none were published prior to 2003, suggesting few, if any, studies published prior to 1990 would have met our inclusion/exclusion criteria. The titles were screened by the first author. All abstracts and full-texts that were not excluded at the title screening stage (*n* = 206) were independently double-screened. There was 100% agreement between the two reviewers regarding the studies to be included.

### Search terms

Perseverative cognition terms (adapted from Querstret and Cropley, 2013; Ottaviani et al., 2015) combined with OR:

(1) perseverati^*^ AND cogniti^*^ (2) reflection (3) brooding (4) ruminat^*^ (5) reflect^*^ AND thought^*^ OR thinking (6) brood^*^ AND thought^*^ OR thinking (7) perseverative AND thought^*^ OR thinking (8) repetitive AND thought^*^ OR thinking (9) intrusive AND thought^*^ OR thinking (10) negative AND thought^*^ OR thinking (11) self-referential AND thought^*^ OR thinking (12) stress AND thought^*^ OR thinking (13) obsessive AND thought^*^ OR thinking (14) worry (15) unconscious stress^*^ (16) implicit stress^*^ (17) anticipat^*^ stress^*^ (17) cognitive intrusion^*^

To increase the specificity of the search strategy, we removed terms used by Querstret and Cropley (2013) which related to anxiety, depression, and stress as, although these concepts overlap with perseverative cognition, they are not specific to perseverative cognition. Aspects of perseverative cognition which do relate to stress and anxiety should be captured by terms such as “stress” combined with “thought^*^ or thinking” and depressive thoughts should be captured by “brooding” and/or “ruminat^*^.” The bulk of the search terms were derived from Querstret and Cropley (2013) and therefore the only term relating to perseverative cognition taken from Ottaviani et al. (2015) was “self-referential” as all of the other relevant terms in this review had already been covered.

Health behavior terms (alcohol terms adapted from Kaner et al., 2007; exercise from Foster et al., 2005; eating from Nield et al., 2007; smoking from Secker-Walker et al., 2002; and sleep from Hu et al., 2015) combined with OR:

(1) exp alcohols/ (2) Alcohol$.tw. (3) exercise.sh. (3) physical activity.sh (4) sports.sh (5) dance.sh (6) [physical$ adj5 (fit$ or train$ or activ$ or endur$)].tw. (7) [exercis$ adj5 (train$ or physical$ or activ$)].tw. (8) sport$.tw. (9) walk$.tw. (10) bicycle$.tw. (11) (exercise$ adj aerobic$).tw. (12) [(lifestyle or life-style) adj5 (activ$)].tw. (13) [(lifestyle or life-style) adj5 physical$].tw. (14) Diets.sh (15) Eating behavio?r.sh (16) weight control.sh (17) (diet$ adj5 carbohydrat$).tw (18) (diet$ adj5 fat$).tw (19) (diet$ adj5 weigh$).tw (20) (diet$ adj5 sugar$).tw (21) (diet$ adj5 fiber$).tw (24) (diet$ adj5 fiber$).tw (22) (diet$ adj5 salt$).tw (23) (diet$ adj5 calorie$).tw (24) healthy eating.tw (25) smok$.mp. (26) nicotine.mp. (27) tobacco.mp. (28) cigarette$.mp. (29) exp sleep/ (30) sleep adj3 (promot^*^ or help^*^ or support^*^ or initiat^*^).mp. (31) sleep.ti,ab

Alcohol terms were not changed from the source but were the only terms relating to alcohol consumption from a larger number of search terms. The same strategy of selecting relevant terms was used in regards to physical activity, diet, smoking and sleep terms. Eating terms were removed which referred to diabetes as this was not relevant to the current review.

The items below were developed by the research team as they were not captured by the terms adapted from the previous reviews cited:

(32) hypophagi^*^ (33) hyperphagi^*^ (34) caffein^*^ (35) snack^*^ (36) meal^*^ (37) junk food^*^ (38) fast food^*^ (39) vegetable^*^ (40) fruit^*^ (41) unhealthy food^*^ (42) unhealthy diet (43) healthy food^*^ (44) alcohol^*^ intake (45) alcohol^*^ unit (46) alcohol^*^ consum^*^ (47) caffein^*^.

Adding these terms increased the number of papers retrieved and ensured that potentially relevant papers were not missed. Perseverative cognition and health behavior terms were then combined with AND.

### Data extraction

The following data were extracted (see Table 1) by the lead author for each study: lead author name, publication year, study design, geographical location, study setting, behavioral outcome (and whether this measure had been previously validated), the type of PC (and whether this measure had been previously validated), the measure of PC, the number of participants included in the analyses, the percentage of female participants and the age of participants (preferably the mean and SD if reported). To maximize reliability of the data extraction process, each section of the data extraction sheet was checked by the co-authors of this paper. Each co-author took responsibility for checking different sections of the data extraction form.

**Table 1:** **Overview of included studies**.

**Lead authors, year**	**Design**	**Location**	**Setting**	**Behavioral domain**	**Type of PC**	**Measure of PC**	**Pps included in Analysis (n)**	**% Female**	**Age of Pps (years)**
Adrian et al., 2014	Prospective	US	School	Substance use	Rumination (brooding and reflection)	RRS^a^	428	48%	12–16
Aldridge-Gerry et al., 2011	Daily diary	US	University	Alcohol consumption	Emotional rumination	Factor analysis Roesch et al., 2010 of Brief COPE, children's coping strategies checklist & how i coped under pressure scale to produce emotional rumination factor	365	69%	Mean = 20 (*SD* = 2)
Bernat et al., 2015	Cross-sectional	US	University	Physical activity	Dispositional cancer worry	Brief worry scale & revised impact of events, intrusive thoughts subscale	451	100%	Mean = 20 (*SD* = 3)
Ciesla et al., 2011	Cross-sectional	US	University	Alcohol consumption	Rumination, angry rumination and worry	RRS, angry rumination scale and PSWQ^b^	447	65%	80% 18–20, 10% 21–25, 2% 25+
Cropley et al., 2012	Cross-sectional	UK	Workplace	Eating	Rumination	Measure of “switching off from work”	268	59%	Mean = 37 (*SD* = 13)
Dijkstra and Brosschot, 2003	Prospective	Netherlands	Home	Smoking cessation	Health worry^*^	4 items developed (worry about the physical consequences of smoking)	704 (380 smokers, 324 ex-Smokers)	Smokers (71%); Ex-Smokers (67%)	Smokers (16–80, mean = 44); Ex-Smokers (15–78, mean = 45)
Dvorak et al., 2011	Cross-sectional	US	Online	Smoking cessation^*^	Rumination	Depressive rumination subscale of RRS	53	79%	Mean = 20 (*SD* = 3)
Ferrer et al., 2013a	Cross-sectional	US	Home	Eating	Health-related worry^*^	1 item developed (worry about overall health in past year)	3397	52%	31% 18–34, 36% 35–54, 33% 55+
Ferrer et al., 2013b	Cross-sectional	US	Home	Eating and physical activity^*^	Cancer-related worry^*^	1 item developed (worry about cancer)	10,230	52%	Mean = 45 (*SD* = 0.06)
Frone, 2015	Cross-sectional	US	Home	Alcohol consumption	Rumination^*^	Negative and positive work rumination scale developed^**^	2831	47%	Mean = 41
Harwell et al., 2011	Cross-sectional	US	University	Alcohol consumption	Anxious rumination	Anxiety rumination questionnaire adapted from rumination on sadness scale	113	82%	Mean = 26
Li et al., 2009	Prospective	US	Home	Physical activity	Health worry^*^	1 item developed (worry about health in past year)	7527	62%	Mean = 77 (*SD* = 6)
Malmi et al., 2010	Case-control	Finland	Home	Prostate cancer screening (objective measure)	Worry^*^	3 items developed (worry about urinary continence and bowel and sexual function)	423	0%	Attended Screening (mean = 60, *SD* = 4); Non-Attenders (mean = 61, *SD* = 5)
Rutten et al., 2011	Cross-sectional	US	Home	Smoking^*^	Cancer worry^*^	2 items developed (worry about lung cancer and fear of screening)	1765 (918 never smoked, 524 former smokers, 323 current smokers)	By smoking status: Never smoked (61%); Former smokers (47%); Current Smokers (46%)	Never Smoked (35% 18–34, 30% 35–49, 21% 50–64, 14% 65+); Former smokers (13% 18–34, 27% 35–49, 34% 50–64, 26% 65+); Current Smokers (39% 18–34, 34% 35–49, 19% 50–64, 8% 65+)
Shoal et al., 2005	Prospective	US	University	Substance use	Worry	6 items from STAI^c^ assessing intrusive thought and worries items from CBCL^d^ (combined to form worry measure)	257	0%	T1 (mean = 11, *SD* = 1); T2 (mean = 16, *SD* = 1)
Swayampakala et al., 2013	Longitudinal	Mexico	Home	Smoking^*^	Health worry^*^	1 item developed (worry about whether smoking will damage health)	1206	32%	18 or over
Willem et al., 2011	Cross-sectional	Belgium	School	Substance use	Rumination (brooding and reflection)	RRS	189	50%	Mean = 17 (*SD* = 1)
Willem et al., 2014	Longitudinal	Belgium	School	Substance use	Rumination (brooding and reflection)	RRS	216	38%	Mean = 17 (*SD* = 1)
Yong et al., 2014	Longitudinal cohort	Australia, Canada, UK, US	Home	Smoking cessation	Health Worry^*^	Items not described (worry about damage from smoking)	5065	By age group: 18–24 (89%), 25–39 (68%), 40–54 (66%), 55+ (77%)	18 or over

* measure not validated (or at least one measure not validated if more than one measure of PC or health behavior),

** only the negative scale was analyzed here.

a Ruminative Responses Scale (Nolen-Hoeksema, 1991),

b Penn State Worry Questionnaire (Meyer et al., 1990),

c State Trait Anxiety Inventory (Spielberger et al., 1983),

d Child Behavior Checklist (Achenbach and Edelbrock, 1983).

### Data synthesis

Comprehensive Meta-Analysis (Borenstein et al., 2005) was used to calculate effect sizes reflecting the relationship between measures of PC and measures of health behaviors. Effect sizes were calculated based on correlation co-efficients and, when not available, were based on other statistical information (e.g., beta or *p*-values). Effect sizes were meta-analyzed within studies when necessary (e.g., when the same variables were assessed at multiple time-points; when different measures of the same behavior were taken in the same study etc.). Effect sizes were combined across studies, where appropriate, using random effect models (where each study estimates different underlying effect sizes) rather than fixed effects models (where all studies are assumed to be estimates of the same one true effect size) because (1) we assumed that the true effect should vary across studies because they differ in critical ways (e.g., type of behavior; type of PC) and (2) our sample of studies, selected systematically, should reflect a random sample of the relevant distribution of effects.

After considering the overall association between PC (worry and rumination) and health behaviors, additional analyses were conducted to identify the association between different types of health behavior (health promoting and health risk) and different types of PC (rumination; worry-health; general worry; plus, the related adaptive construct of reflection). In most instances, formal moderation analyses were not conducted because there were studies in which the same participants completed multiple measures (e.g., participants in the study by Cropley et al., 2012, completed measures of health promoting and health risk behaviors; the participants in the study by Ciesla et al., 2011, completed measures of rumination and worry). In terms of the worry measures, it is worth noting that a number of studies included a measure of health-specific worry (e.g., worry about overall health in the past year, or worry about developing cancer) which is distinct from general worry (e.g., I worry too much about making mistakes, about my parents, about things that may happen, and about what others think of me).

Sensitivity analyses were conducted to examine if the results changed when measures that related to quit attempts were removed (given its qualitative difference from standard measures of performing health behaviors; sensitivity analysis 1) or when other types of unique measure were removed (sensitivity analysis 2 excluded the study by Harwell et al., 2011, given they only considered drinking in negative situations rather than drinking across all situations, and removed the measure of affect-related substance use from the effect size calculation for Shoal et al., 2005, for similar reasons). In the case of quit attempts, we felt it was unclear whether a high number of quits is positive (indicative of greater desire to stop smoking) or negative (indicative of more failed attempts). It is also not a clear measure of health behavior (in the same sense as the other measures included); it could be argued to be a measure of “trying” or “motivation.” Therefore, we felt it was appropriate to examine in our sensitivity analyses.

In all analyses, a positive correlation reflects an association between increased levels of PC and increased unhealthy behavior (i.e., either more health-risk behavior or less health promoting behavior). A negative correlation reflects an association between increased levels of PC and increased healthy behavior (i.e., either less health-risk behavior or more health promoting behavior).

## Results

### Overview of included studies

The search returned 7504 papers which were screened for inclusion. Screening identified 19 relevant studies (see Figure 2 and Table 1). Of the 19 included studies, 9 measured rumination (emotional rumination: Aldridge-Gerry et al., 2011; rumination: Ciesla et al., 2011; Dvorak et al., 2011; Willem et al., 2011; Cropley et al., 2012; Adrian et al., 2014; Frone, 2015; Willem et al., 2014; angry rumination: Ciesla et al., 2011; anxious rumination: Harwell et al., 2011), 9 studies measured health-related worry (Dijkstra and Brosschot, 2003; Li et al., 2009; Malmi et al., 2010; Rutten et al., 2011; Ferrer et al., 2013a,b; Swayampakala et al., 2013; Yong et al., 2014; Bernat et al., 2015), and 2 studies measured general worry (Shoal et al., 2005; Ciesla et al., 2011). In addition, four studies measured reflection (Willem et al., 2011, 2014; Cropley et al., 2012; Adrian et al., 2014). Note that Ciesla et al. (2011) also measured co-rumination but this was removed as our conceptualization of rumination did not include this and the Cropley et al. (2012) measure of problem-solving pondering was classified as reflection in our analyses.

Health behaviors investigated were alcohol consumption (Shoal et al., 2005; Aldridge-Gerry et al., 2011; Ciesla et al., 2011; Harwell et al., 2011; Willem et al., 2011, 2014; Adrian et al., 2014; Frone, 2015), marijuana use (Shoal et al., 2005; Willem et al., 2011; Adrian et al., 2014; Willem et al., 2014), smoking behavior and cessation (Dijkstra and Brosschot, 2003; Dvorak et al., 2011; Rutten et al., 2011; Swayampakala et al., 2013; Yong et al., 2014), eating behavior (Cropley et al., 2012; Ferrer et al., 2013a,b), cancer screening uptake (Malmi et al., 2010) and levels of physical activity (Li et al., 2009; Ferrer et al., 2013b; Bernat et al., 2015). See Table 1 for a more detailed overview of the included studies. Table 2 presents the results of the meta-analyses.

**Table 2:** **Summary of meta-analyses**.

**Type of PC**	**Health Behavior**	***k***	***R***	**95% CI**		***Z***	**Sensitivity Analyses: *Z***	
				**Lower**	**Upper**		**Analysis 1**	**Analysis 2**
All	All	19	0.066	−0.015	0.147	1.599	2.493^*^	1.987^*^
Rumination	All	9	0.103	0.046	0.160	3.527^***^	3.371^**^	4.886^***^
Reflection	All	4	−0.008	−0.074	0.058	−0.231	-	-
Worry (all)	All	11	0.013	−0.096	0.122	0.238	0.864	0.850
Worry (health)	All	9	0.019	−0.111	0.148	0.286	0.895	0.895
Worry (other)	All	2	−0.002	−0.142	0.139	−0.021	-	−0.002
All	Health promotion	6	−0.038	−0.101	0.025	−1.181	-	-
All	Health risk	15	0.106	0.005	0.205	2.055^*^	3.564^***^	3.160^**^
Rumination	Health promotion	1	0.000	−0.085	0.085	0.000	-	-
Rumination	Health risk	9	0.122	0.058	0.184	3.758^***^	3.606^***^	3.932^***^
Reflection	Health promotion	1	−0.080	−0.198	0.040	−1.305	-	-
Reflection	Health risk	4	0.012	−0.061	0.085	0.320	-	-
Worry (all)	Health promotion	5	−0.045	−0.120	0.030	−1.188	-	-
Worry (all)	Health risk	7	0.048	−0.113	0.207	0.585	1.495	1.432

k, number of studies; r, effect size r; 95% CI, 95% confidence interval of effect size r;

* p <0.05,

** p <0.01,

*** p <0.001; Sensitivity Analysis 1: Quit attempts for smoking are excluded from the analyses; Sensitivity Analysis 2: Excludes quit attempts for smoking, Harwell et al. (2011) (negative reinforcement drinking), Shoal et al. (2005) (affect-related substance use measure removed but measure of drug use still incorporated). In the sensitivity analyses, “-“ indicates the results match the original analyses.

### Main results

Averaging across all types of PC (rumination and worry), behaviors and time-points, PC was initially unrelated with health behaviors, *r* = 0.066, 95% *CI* = −0.015 to 0.147, *Z* = 1.599, *p* = 0.110, with very heterogeneous effect sizes, *Q*_(18)_ = 324.562, *p* <0.001, *I*^2^ = 94.454 (see Table 2). However, in the sensitivity analyses, the relationship between PC and health behaviors became significant, albeit still small. Specifically, more PC was associated with unhealthier behaviors (a combination measure of more health risk behaviors/fewer health promoting behaviors), *r* = 0.079, 95% *CI* = 0.017–0.140, *Z* = 2.493, *p* = 0.013 (sensitivity analysis 1), *r* = 0.057, 95% *CI* = 0.001–0.113, *Z* = 1.987, *p* = 0.047 (sensitivity analysis 2).

### PC type

Increases in rumination were associated with unhealthier behaviors (combination measure of more health risk behaviors/fewer health promoting behaviors), *r* = 0.103, 95% *CI* = 0.046–0.160, *k* = 9, *Z* = 3.527, *p* <0.001. Reflection, *r* = −0.008, 95% *CI* = −0.074 to 0.058, *k* = 4, *Z* = −0.231, *p* = 0.817, and worry, *r* = 0.013, 95% *CI* = −0.096–0.122, *k* = 11, *Z* = 0.238, *p* =.812, were unrelated with health behaviors. The heterogeneity in effect sizes were particularly large for the studies that included a measure of worry, *Q*_(10)_ = 217.972, *p* <0.001, *I*^2^ = 95.412. Comparing the studies that incorporated a measure of worry related to health (*k* = 9) against those that included an alternative measure of worry (*k* = 2), based on random effects models, the effects were similar (health-related worry and health behaviors: *r* = 0.019, 95% *CI* = −0.111 to 0.148, *Z* = 0.286, *p* = 0.775; other worry and health behaviors: *r* = −0.002, 95% *CI* = −.142 to 0.139, *Z* = −0.021, *p* = 0.983; *Q*_(1)_ = 0.044, *p* = 0.834. The results of the sub-group analyses, split by PC type, were influenced little by the sensitivity analyses.

### Type of behavior

PC was unrelated to health promoting behaviors but was significantly related with health risk behaviors. Regarding the latter, increases in PC were associated with increased performance of health risk behaviors. These relationships were consistent across both sets of sensitivity analyses (see Table 2).

### PC type and behavior

Increases in rumination were associated with increased performance of health risk behavior but not health promoting behavior (though only one study, Cropley et al., 2012, has considered the latter association). Worry and reflection were both unrelated to health promoting and health risk behaviors (though only one study, Cropley et al., 2012, considered the association between reflection and health promoting behaviors). These results did not change substantively in either set of sensitivity analyses.

### Publication bias

Egger's regression coefficient was significant for the relationship between PC and health behaviors (combination of health risk and health promotion behaviors; *p* = 0.005) suggesting some degree of publication bias. To consider the potential impact of these missing studies, Duval and Tweedie's Trim and Fill analyses were conducted. These results suggested that no studies were missing from the left-side of the mean effect but six studies were missing from the right-side of the mean effect. After imputing these, the imputed point estimate, *r* = 0.142, 95% *CI* = 0.033–0.248, suggested, if anything, that the relationship between PC and unhealthy behaviors is slightly stronger than estimated in the main analyses.

## Discussion

The main findings of this systematic review and meta-analysis are that increases in PC are associated with increases in health risk behaviors (substance use, alcohol consumption, unhealthy eating, and smoking) that are driven primarily through rumination. In contrast, measures of worry and reflection were not associated with health behaviors. These results are important for a number of reasons. First, they provide partial support for our hypothesis that in Brosschot et al.'s (2006) original PCH, there may be scope for an additional route to pathogenic disease via poorer health behaviors. In this conceptualization, we theorize that rumination about past stressful events will mediate the effects of stressors on health behaviors (particularly those previously shown to be influenced by stress), which will have negative consequences for health outcomes and disease processes.

Second, from a brain-body point of view, the current findings are important in the context of the development of **allostatic load**. McEwen (1998) introduced the concept of allostatic load to capture the wear and tear the body experiences as a result of repeated and prolonged adaption to environmental and psychosocial stressors. He proposed that the long-term impact of stress affects the body at cardiovascular, metabolic, neural, behavioral, and cellular levels. Similar to basic homeostatic systems such as body temperature, the **HPA axis**, the autonomic nervous system and the cardiovascular, metabolic and immune systems protect the body by adapting to internal and external stress. This is known as **allostasis**. However, if the activation of these systems (allostasis) is repeated and prolonged, allostatic load will be experienced in the form of increased stress hormone, immune cell, brain activity, and cardiovascular responses, ultimately, overtime leading to heightened risk of developing disease (McEwen, 1998, 2007). Numerous factors may contribute to the development of allostatic load including genes, early life experiences and disturbances of the sleep-wake cycle (McEwen, 2007). However, McEwen also argues that lifestyle choices such as alcohol consumption, diet, smoking, and exercise, that may be learned overtime (and triggered by PC), contribute to allostatic load by influencing the reactivity of the biological systems that release the physiological stress mediators (e.g., cortisol, adrenaline, blood pressure, heart rate, immune cells). In other words, environmental and psychosocial stressors give rise to PC, which in turn triggers maladaptive behavioral responses that may influence and exacerbate the prolonged stress response as conceptualized in the PCH, leading to increased risk of disease. Moreover, we also contend that the relationship between the prolonged stress response and health behaviors may be bi-directional (see Figure 1). Interestingly, in the short term, engaging in health risk behaviors such as comfort eating or alcohol consumption (triggered by stressors and then PC) may be perceived by individuals as beneficial, however, overtime these behaviors are likely to be damaging for health.

In addition to the PC-induced health behavior-prolonged stress response pathway, it is highly likely that PC-induced health risk behaviors will directly impact on pathogenic states such as changes in somatic health outcomes (see second dashed pathway in Figure 1). For example, for eating behavior, it is well established that stress (and possibly PC) contributes directly to diseases like cardiovascular disease and obesity risk to the extent that it produces deleterious changes in diet and helps maintain unhealthy eating behaviors (O'Connor et al., 2015). In terms of physical activity, a recent systematic review and meta-analysis, showed that greater time spent sedentary was linked to increased risk of diabetes, cardiovascular events, cardiovascular mortality, and all-cause mortality (Wilmot et al., 2012). Therefore, again the extent to which PC can disrupt habitual health behaviors such as exercise, eating behavior, alcohol consumption, and smoking, is likely to increase its direct effects on behavioral mediated changes in pathogenic states.

Nevertheless, we recognize that the current results ought to be considered preliminary at this stage precluding any firm conclusions. We are mindful that our analyses did not find evidence that worry about feared future events was associated with health behaviors. This is surprising given that worry has been identified as important in recent narrative reviews and meta-analyses in the context of the PCH (Verkuil et al., 2010; Ottaviani et al., 2015). A likely explanation for the absence of a significant effect here might be related to the heterogeneity of effect sizes across the studies and/or to do with the variability in types of worry measures utilized (e.g., health-related worry, cancer worry, trait worry, etc. as well as single-item vs. multi-item measures). Alternatively, this null finding may reflect that there are relatively few studies that have directly investigated the relationship between worry (and rumination) and health risk and health promoting behaviors. In many of the studies reviewed, exploring the relationship between worry (and rumination) has been of secondary interest. It might also be that worry, triggered by fear-appeals, has the capacity to promote some health behaviors, thereby, contributing to the observed mixed findings (Tannenbaum et al., 2015). We also acknowledge that limiting our search strategy to studies published from 1990 onwards may have resulted in our missing some important studies. However, we feel the potential impact of this approach is likely to be fairly minimal given that of the articles included in our review, none were published prior to 2003, suggesting few, if any, studies published prior to 1990 would have met our inclusion/exclusion criteria.

We hope the current findings will spur on PC researchers to include measures of health behaviors in their future studies and to adopt a myriad of different approaches to investigate the precise processes and mechanisms through which PC is linked to health behaviors in the context of the PCH. It is likely that these processes will differ dependent on the nature of the health behavior (i.e., health risk vs. health promoting, frequency of the behavior etc.) and in relation to the type of PC (rumination vs. worry; health-related worry vs. general worry etc.). Future research ought to attempt to replicate the current findings utilizing innovative techniques such as ecological momentary assessment, diary methods and time-lagged designs in combination with measures of the physiological concomitants of PC (Verkuil et al., 2012; Gartland et al., 2014). There is also scope to manipulate PC in carefully controlled laboratory studies in order to investigate whether changes in PC are associated with changes in health behaviors (such as food intake; cf., Newman et al., 2007).

In conclusion, this systematic review and meta-analysis showed that increases in PC are associated with increases in health risk behaviors (substance use, alcohol consumption, unhealthy eating, and smoking) that are driven primarily through rumination. These findings provide partial support for our hypothesis that in Brosschot et al.'s (2006) original PCH, there may be scope for an additional route to pathogenic disease via poorer health behaviors.

**Table:** 

**Key Term**	**Definition**
Worry	Negative, repetitive cognitions regarding feared future events
Rumination	Negative, repetitive thoughts regarding feelings and problems (past-focused)
Perseverative Cognition	Negative, repetitive, cognitive representations of past stressful events or feared future events
Allostatic Load	The wear and tear the body experiences as a result of repeated and prolonged adaption to environmental and psychosocial stressors
Allostasis	When the autonomic nervous system and the cardiovascular, metabolic and immune systems protect the body by adapting to internal and external stress
Hypothalamic-Pituitary-Adrenal-Axis (HPA Axis)	Biological feedback loop between the hypothalamus, pituitary gland and adrenal glands which controls the body's stress response

## Author contributions

DO, FC, and AP conceived of the systematic review and meta-analysis. FC and LC conducted the data extraction and coding with input from AP and DO. AP and FC performed the meta-analysis. DO, AP, and FC drafted the manuscript. All authors approved the final version and agree to be accountable for this work.

## Funding

FC is funded by an ESRC White Rose Training Centre Advanced Quantitative Methods Ph.D. studentship.

### Conflict of interest statement

The authors declare that the research was conducted in the absence of any commercial or financial relationships that could be construed as a potential conflict of interest.
